# Cultural adaptation and validation of the Health Literacy Questionnaire (HLQ): robust nine-dimension Danish language confirmatory factor model

**DOI:** 10.1186/s40064-016-2887-9

**Published:** 2016-08-02

**Authors:** Helle Terkildsen Maindal, Lars Kayser, Ole Norgaard, Anne Bo, Gerald R. Elsworth, Richard H. Osborne

**Affiliations:** 1 Department of Public Health, Section of Health Promotion and Health Services Research, Aarhus University, Bartholins Alle 2, 8000 Aarhus C, Denmark; 2 Steno Diabetes Center, Health Promotion Research, Gentofte, Denmark; 3 Department of Public Health, University of Copenhagen, Copenhagen, Denmark; 4 Research and Knowledge Centre, Danish Veteran Centre, Ringsted, Denmark; 5 Health Systems Improvement Unit, Deakin University Centre for Population Health Research, School of Health and Social Development, Geelong, VIC Australia

**Keywords:** Health literacy, Questionnaire, Measurement, Validation, Psychometrics, HLQ

## Abstract

Health literacy is an important construct in population health and healthcare requiring rigorous measurement. The Health Literacy Questionnaire (HLQ), with nine scales, measures a broad perception of health literacy. This study aimed to adapt the HLQ to the Danish setting, and to examine the factor structure, homogeneity, reliability and discriminant validity. The HLQ was adapted using forward–backward translation, consensus conference and cognitive interviews (n = 15). Psychometric properties were examined based on data collected by face-to-face interview (n = 481). Tests included difficulty level, composite scale reliability and confirmatory factor analysis (CFA). Cognitive testing revealed that only minor re-wording was required. The easiest scale to respond to positively was ‘Social support for health’, and the hardest were ‘Navigating the healthcare system’ and ‘Appraisal of health information’. CFA of the individual scales showed acceptably high loadings (range 0.49–0.93). CFA fit statistics after including correlated residuals were good for seven scales, acceptable for one. Composite reliability and Cronbach’s α were >0.8 for all but one scale. A nine-factor CFA model was fitted to items with no cross-loadings or correlated residuals allowed. Given this restricted model, the fit was satisfactory. The HLQ appears robust for its intended application of assessing health literacy in a range of settings. Further work is required to demonstrate sensitivity to measure changes.

## Background

The complexity of modern healthcare and the many health messages being promoted have led to health literacy being a key consideration for health promotion and improving the quality of health services (Nutbeam 2000; Protheroe et al. 2009; Sørensen et al. 2012; Kickbusch et al. 2013; Norgaard et al. 2014). Furthermore, the need for people to manage their health themselves, including using various health technologies, requires individuals to have a wide range of health literacy competencies.

The World Health Organization defined health literacy as “the cognitive and social skills which determine the motivation and ability of individuals to gain access to, understand and use information in ways which promote and maintain good health” (World Health Organization 1998). From the consumer’s perspective the required competencies include not only being able to read and understand health information, but also being able to navigate the health system, communicate and engage with healthcare providers, engage in critical appraisal of health information, and advocate for one’s right to health services (HLS-EU Consortium 2012; Kickbusch et al. 2013; Osborne et al. 2013). There is, therefore, great potential to improve public health and clinical medicine by addressing health literacy in organisations on many levels through clinician training, organisational policy and planning, health protection, and public health policy.

In Denmark, and many other countries, there is a need for data about population health literacy in order to cultivate an inclusive health system that meets the needs of individuals and diverse population groups (Sorensen et al. 2014).

The focus of previous health literacy measurement methods was on functional health literacy: word recognition tests, reading ability and numeracy (Mårtensson and Hensing 2012; Haun et al. 2014). Recent instruments, such as the Health Literacy Questionnaire (HLQ) (Osborne et al. 2013) and the European Health Literacy Survey (HLS-EU) (HLS-EU Consortium 2012), assess a wider range of health literacy components. Data derived from the HLQ are designed to assist with the diagnosis of the diverse health literacy needs of individuals and communities in both health promotion and clinical settings. It has nine scales that generate comprehensive profiles of the health literacy of individuals and groups. The profiles then assist practitioners, planners and policy makers to understand the health literacy needs of communities and this in turn assists in planning, designing and evaluating interventions.

The HLQ has been widely translated and applied in research, evaluation and monitoring (Deakin University 2016). However, in order for data from a new measure to be regarded as sufficiently robust to make decisions about individuals, communities or organisations, or to compare across settings, psychometric evidence is required that demonstrates that it is culturally and linguistically appropriate and has strong measurement properties.

In the development of the English language HLQ the items were chosen to best represent the constructs that had been generated through a ‘grounded validity-driven’ approach which included both qualitative and quantitative techniques among both consumers and professionals (Buchbinder et al. 2011). The meaning of the constructs was established by this grounded process and validated by the psychometric analyses reported in the original paper (Osborne et al. 2013). A strict translation protocol was then instituted to ensure that this meaning was carried through accurately to all non-English-language versions. The psychometric analyses in the present paper have sought to replicate the results in the original paper and thus validate the meaning of the Danish scales as equivalent to that established by the original procedures.

The aims of this study were to translate the HLQ to Danish and test the psychometric properties of the translated version in a Danish validation sample. Adaptation was done through a rigorous cultural and linguistic translation procedure, followed by psychometric analyses. The outcome will inform stakeholders and the research community if the Danish HLQ data enable valid and reliable decisions to be made about health literacy in Danish settings.

## Methods

### Setting and data collection

The study was undertaken in primary care settings (community health centres and general practices), public places and workplaces with wide geographic and sociodemographic variation. These settings were consistent with populations and areas in which future application of the HLQ is planned. Data were gathered by health professionals and students by face-to-face interview in both the cognitive testing and validation samples using a standardised protocol.

### Health Literacy Questionnaire (HLQ)

The HLQ was designed using a grounded, validity-driven approach and initially tested in diverse samples of individuals in Australian communities and shown to have strong construct validity, reliability and acceptability to clients and clinicians (Osborne et al. 2013; Batterham et al. 2014). It was designed for administration by pen and paper self-administration or by interview to ensure inclusion of people who cannot read or have other difficulties with self-administration.

The HLQ contains 44 questions that cover nine conceptually distinct areas of health literacy:

1. Feeling understood and supported by healthcare providers (four items).
2. Having sufficient information to manage my health (four items).
3. Actively managing my health (five items).
4. Social support for health (five items).
5. Appraisal of health information (five items).
6. Ability to actively engage with healthcare providers (five items).
7. Navigating the healthcare system (six items).
8. Ability to find good health information (five items).
9. Understand health information well enough to know what to do (five items).

Response options for each scale were determined by the content and nature of the items. For scales 1–5 four-point ordinal response options are used (strongly disagree, disagree, agree and strongly agree), while for scales 6–9 five-point ordinal response options are used (cannot do, very difficult, quite difficult, quite easy and very easy).

### Translation, cultural adaptation, and item strength equivalence

A standardised procedure (Hawkins and Osborne 2010) was used including recommendations from a range of organisations (Guillemin et al. 1993; Wild et al. 2005; Koller et al. 2007). The translation process included an initial discussion of cultural differences between the Australian and Danish health systems and the presence of local dialects in Denmark. It involved two forward translators, two backward translators, the authors, and was chaired by one of the HLQ developers (RHO). The process involved three main steps:

1. Professional translators, with Danish as their native language, were briefed and provided with the English HLQ and detailed information about the intent of each of the nine scale constructs and each of the items within those scales. The item intent document included narratives about precisely what each element of the HLQ items mean and, in some cases, what these do not mean.
2. Back translation was undertaken by two native English speakers with excellent Danish language skills.
3. Finally, a translation consensus meeting was conducted by an expert panel comprising the four translators and four researchers in the fields of population health, health promotion and patient care (HTM), healthcare systems and medicine (ON, LK), and questionnaire development and translation methodology (RHO). Each translated item was examined in turn. Panel members confirmed that: (a) the principal scale construct embodied in each Danish item matched the original English item; (b) the translated items were suitable for males and females, for a wide age range, for people with low linguistic skills, limited experience of health and the healthcare system, and who either did or did not have a health problem; and (c) the relative ‘difficulty’ (in choosing the positive response options agree/strongly agree and quite easy/very easy) of each item was equivalent between the languages. When a word from a Danish dialect was used in the translation, panel members were required to consider how ubiquitous the word is across the country, or if a more commonly-used word with the same meaning would be more appropriate.

The level of comprehensibility and cognitive equivalence of the preliminary translated Danish HLQ (Willis 1999; Wild et al. 2005) was field tested through face-to-face cognitive interviews with 15 persons across gender and education categories and from different parts of Denmark. The cognitive testing involved initial administration of items using paper and pen format with careful observation of each respondent. The interviewer then reviewed items with the respondent and asked specific questions about items they had hesitated on or appeared to have found difficult in answering. Respondents were asked ‘What were you thinking about when you were answering that question?’ This process elicited the cognitive process behind the answers. A prompt was used if needed: ‘Why did you select that response option?’

### Ethics

The study was approved by the Danish Data Protection Agency (j.no: 2013-41-2270). According to Danish law, when survey-based studies are undertaken in accordance with the Helsinki Declaration, specific approval by an ethics committee and written informed consent is not required. Potential respondents were provided with information about the survey and its purpose, including that participation was voluntary. The completion of the survey by participants was then considered to be implied consent.

### Statistical analysis

Analyses were conducted with STATA version 13.0 and 14.0 and Mplus version 7.11. Descriptive statistics were generated for each item to determine the extent of missing values and to demonstrate the range of responses by providing difficulty estimates within and across the nine scales. For scales with disagree/agree response options, the relative strength of an item (how difficult it is to score highly) was calculated as the proportion responding disagree and strongly disagree (low scores) as against agree or strongly agree (high scores). For scales with response options cannot do to very easy, the difficulty level was calculated as the proportion responding cannot do, very difficult, or quite difficult as against quite easy and very easy (Raykov and Marcoulides 2011).

As Cronbach’s α is frequently a biased estimate of population reliability, unbiased estimates of composite reliability were generated (Raykov 2007). However, α was also calculated for comparison with other studies. Given that the HLQ scales were specified a priori, confirmatory factor analysis (CFA) was undertaken. Using one-factor CFA, a model was fitted to the data for each previously confirmed scale (Osborne et al. 2013). The response options were scored as ordinal variables (1–4 for the strongly disagree–strongly agree scales; 1–5 for the cannot do–very easy scales) and the models were fitted using the weighted least squares mean and variance adjusted (WLSMV) estimator available in Mplus. Unstandardised and standardised factor loadings, an estimate of the variance in the measured variable explained by the latent variable (R^2^), and associated standard errors are provided in Mplus together with fit statistics (*χ*^2^, comparative fit index—CFI, Tucker–Lewis index—TLI, and root mean square error of approximation—RMSEA). Indicative threshold values for the tests of ‘close fit’ used in this analysis were CFI > 0.95; TLI > 0.95; RMSEA < 0.06 while a value of <0.08 for the RMSEA was taken to indicate a “reasonable” fit (Browne and Cudeck 1993; Yu 2002; West et al. 2012).

Mplus also provides statistics that can be used to facilitate model improvement by suggesting fixed parameters (e.g., in the case of single-factor models, correlations among residual variances) that might be freely estimated. In Mplus, these statistics include standardised residuals, modification indices (MIs) and the associated change in a parameter if the modification is included in the model (standardised expected parameter change—SEPC).

A full nine-factor CFA model with no correlated residuals or cross-loadings was fitted to the data to investigate discriminant validity. As the restrictions that are typically placed on multi-factor CFA models frequently result in a strong upwards bias in the estimation of inter-factor correlations, this analysis was followed-up by fitting the nine-factor model using Bayesian structural equation modelling (BSEM) (Marsh et al. 2010). By using small-variance priors, BSEM allows models to be fitted that have the flexibility to estimate small variations from the strictly zero constraints on the residual correlations and cross-loadings in a typical multi-factor CFA model (Muthén and Asparouhov 2012). For this analysis, the variance of the Bayesian priors for the cross-loadings was set at 0.02 such that there was a 95 % probability that the cross-loadings would be within the range ±0.28. Similarly, the variance for the residual correlations was set to give a 95 % probability that the correlations were within the range of ±0.2 (Muthén and Asparouhov 2012).

HLQ scale scores were calculated as unit-weighted sums of the constituent items averaged by the number of items in the scale such that the nominal range of the scale scores was 1–4 for scales 1–5 and 1–5 for scales 6–9. One-way ANOVA was used to investigate mean differences on the HLQ scale scores across a range of sociodemographic variables. Effect sizes (ES) and their 95 % confidence intervals (CI) for standardised differences in means between sociodemographic groups were calculated using Cohen’s ‘d’ with interpretation of ES as follows: “small” ES > 0.20–0.50, “medium” ES approximately 0.50–0.80, and “large” ES > 0.80.

## Results

### Cognitive testing

Cognitive testing revealed that almost all items were well understood. Only minor re-wording was required.

### Validation sample characteristics

Table 1 shows the sociodemographic characteristics and health conditions of the 481 respondents in the validation sample. The median age was 53 years, with the youngest being 17, and the oldest 92. There were more women than men (a percentage-point difference of 19.2), while 18.9 % of respondents lived alone. Over 50 % had completed a high school or higher and 60 % reported having a longstanding illness or disability. The most frequent chronic conditions reported were: musculoskeletal disorders, cardiovascular disease, diabetes, cancer, chronic obstructive pulmonary disease and mental disorders. Just over a quarter of respondents lived in the capital area of Denmark and 63 % lived in other Danish cities across different geographical regions.

**Table 1:** Characteristics of participants in the validation of the Health Literacy Questionaire (HLQ), Danish version (n = 481)

Characteristics		n
Age, years median (IQR)	53 (36–62)	449
Sex Female [n (%)]	286 (59.6)	480
Mother tongue Danish [n (%)]	445 (92.9)	479
Living area Copenhagen (capital) [n (%)]	118 (26.8)	441
Education level [n (%)]		479
Still in school	10 (2.1)	
7 or fewer years of school	34 (7.1)	
8–9 years of school	47 (9.8)	
10–11 years of school	120 (25.1)	
High school diploma	247 (51.6)	
Other	21 (4.4)	
Cohabitation Live alone [n (%)]	90 (18.9)	475
Self-reported health status [n (%)]		481
Excellent	97 (20.2)	
Very good	169 (35.1)	
Good	156 (32.4)	
Less good	52 (10.8)	
Poor	7 (1.5)	
Long-standing illness or disability, yes	288 (59.9)	
Self-reported chronic disease [n (%)]^a^		
Diabetes	50 (10.5)	477
Chronic Obstructive Pulmonary disease	39 (8.2)	477
Cardiovascular disease (stroke, angina pectoris, AMI)	124 (26.1)	475
Cancer	43 (9.1)	475
Musculoskeletal (arthritis, osteoporosis, back pain or other)	165 (34.9)	473
Mental health condition >6 months	38 (8.0)	476

^a^More than one possible

### Psychometric properties of the Danish HLQ

Response to the HLQ items was high (missing answers: 0.2–1.7 %). Tables 2 and 3 present the psychometric properties of the items and scales. At the item level, there were few missing data, with the highest proportion being for item 7.6 ‘Work out what is best care for you’ (1.7 %). The relative strength of the items within scales and between scales varied modestly. The scales that were easiest to score highly were 4. ‘Social support for health’ (average item difficulty 0.14) and 2. ‘Having sufficient information to manage my health’ (0.16). The scales that were hardest to score highly were 7. ‘Navigating the healthcare system’ (0.36) and 5. ‘Appraisal of health information’ (0.36). The easiest item was found in scale 4. ‘Social support for health’ [4.4 ‘I have at least one person who can come to medical appointments with me’ (0.07)], and the hardest item was in scale 7. ‘Navigating the healthcare system’ [7.5 ‘Find out what healthcare services you are entitled to’ (0.51)]. The scale with the smallest range of difficulty was 8. ‘Ability to find good health information’ (hardest 0.21, easiest 0.15, range 0.06), whereas 1. ‘Feeling understood and supported by healthcare providers’ had the largest range of difficulties (hardest 0.36, easiest 0.11, range 0.25).

**Table 2:** Data quality of the translated Health Literacy Questionnaire (HLQ) in a Danish population (n = 481)

Subscale/items	Obs (n = 481)	Missing [n (%)]	Median	Mean (SD)	Strongly disagree (%)	Disagree (%)	Agree (%)	Strongly agree (%)	*Difficulty level (%) (95 % CI)
1. Feeling understood and supported by healthcare providers									
I have at least one healthcare provider who knows me well …	479	2 (0.4)	3	2.79 (0.89)	8.4	27.1	41.5	23.0	35.5 (31.3–39.9)
I have at least one healthcare provider I can discuss …	478	3 (0.6)	3	2.98 (0.75)	4.0	16.7	56.3	23.0	20.7 (17.3–24.6)
I have the healthcare providers I need to help me work …	480	1 (0.2)	3	3.13 (0.61)	1.0	9.8	64.0	25.2	10.8 (8.3–14.0)
I can rely on at least one healthcare provider …	477	4 (0.8)	3	3.08 (0.68)	2.1	13.0	59.7	25.2	15.1 (12.1–18.6)
				3.00 (0.60)					20.5
2. Having sufficient information to manage my health									
I feel I have good information about health	478	3 (0.6)	3	3.15 (0.61)	1.3	8.8	64.0	25.9	10.0 (7.6–13.1)
I have enough information to help me deal with my …	478	3 (0.6)	3	3.09 (0.68)	2.3	11.9	60.5	25.3	14.2 (11.4–17.7)
I am sure I have all the information I need to manage …	478	3 (0.6)	3	3.01 (0.69)	3.1	13.4	62.6	20.9	16.5 (13.5–20.1)
I have all the information I need to look after my health	478	3 (0.6)	3	2.98 (0.68)	1.5	19.7	58.6	20.3	21.1 (17.7–25.0)
				3.06 (0.55)					15.5
3. Actively managing my health									
I spend quite a lot of time actively managing my health	476	5 (1.1)	3	2.72 (0.75)	4.0	33.6	48.5	13.9	37.6 (33.3–42.1)
I make plans for what I need to do to be healthy	476	5 (1.1)	3	2.79 (0.79)	5.0	28.8	48.1	18.1	33.8 (29.7–38.2)
Despite other things in my life, I make time to be healthy	478	3 (0.6)	3	2.62 (0.74)	4.0	41.8	42.3	11.9	45.8 (41.4–50.3)
I set my own goals about health and fitness	479	2 (0.4)	3	3.00 (0.67)	1.7	17.5	60.3	20.5	19.2 (15.9–23.0)
There are things that I do regularly to make myself more healthy	480	1 (0.2)	3	3.04 (0.68)	2.3	14.0	60.8	22.9	16.3 (13.2–19.8)
				2.84 (0.55)					30.5
4. Social support for health									
I can get access to several people who understand and …	478	3 (0.6)	3	3.10 (0.67)	1.7	12.8	59.4	26.2	14.4 (11.6–17.9)
When I feel ill, the people around me really understand …	478	3 (0.6)	3	2.90 (0.72)	4.2	18.8	59.6	17.4	23.0 (19.4–27.0)
If I need help, I have plenty of people I rely on	479	2 (0.4)	3	3.17 (0.72)	2.3	11.9	52.2	33.6	14.2 (11.3–17.6)
I have at least one person who can come to medical …	479	2 (0.4)	3	3.31 (0.66)	2.3	4.4	53.2	40.1	6.7 (4.8–9.3)
I have strong support from family and friends	480	1 (0.2)	3	3.21 (0.65)	1.5	8.5	57.3	32.7	10.0 (7.6–13.0)
				3.14 (0.52)					13.7
5. Appraisal of health information									
I compare health information from different sources	479	2 (0.4)	3	2.85 (0.75)	5.2	21.5	56.6	16.7	26.7 (22.9–30.9)
When I see new information about health, I check up …	477	4 (0.8)	3	2.62 (0.80)	6.7	38.4	41.5	13.4	45.1 (40.6–49.6)
I always compare health information from different sources …	477	4 (0.8)	3	2.69 (0.80)	5.2	35.8	43.2	15.7	41.1 (36.7–45.6)
I know how to find out if the health information I receive is …	476	5 (1.1)	3	2.91 (0.75)	4.2	21.0	54.8	20.0	25.2 (21.5–29.3)
I ask healthcare providers about the quality of the …	479	2 (0.4)	3	2.68 (0.79)	6.1	34.2	45.5	14.2	40.3 (36.0–44.8)
				2.75 (0.56)					35.7

Subscale/items	Obs (n = 481)	Missing n (%)	Median	Mean (SD)	Cannot do (%)	Very difficult (%)	Quite difficult (%)	Quite easy (%)	Very easy (%)	*Difficulty level (%) (95 % CI)
6. Ability to actively engage with healthcare providers										
Make sure that healthcare providers understand your …	479	2 (0.4)	4	3.79 (0.75)	0.8	3.5	24.8	57.2	13.6	29.2 (25.3–33.5)
Feel able to discuss your health concerns with a …	480	1 (0.2)	4	4.11 (0.72)	0.4	2.7	10.2	58.3	28.3	13.3 (10.6–16.7)
Have good discussions about your health with doctors	479	2 (0.4)	4	4.05 (0.81)	1.0	3.5	13.2	53.7	28.6	17.7 (14.6–21.4)
Discuss things with healthcare providers until you understand …	477	4 (0.8)	4	3.98 (0.78)	1.0	2.9	16.8	55.8	23.5	20.8 (17.3–24.6)
Ask healthcare providers questions to get the …	477	4 (0.8)	4	4.00 (0.74)	0.6	2.3	16.4	57.7	23.1	19.3 (16.0–23.1)
				3.99 (0.59)						20.1
7. Navigating the healthcare system										
Find the right healthcare	479	2 (0.4)	4	3.58 (0.86)	3.3	5.8	29.0	52.6	9.2	38.2 (33.9–42.7)
Get to see the healthcare providers I need to	478	3 (0.6)	4	3.72 (0.85)	1.0	7.3	25.9	49.8	15.9	34.3 (30.2–38.7)
Decide which healthcare provider you need to see …	478	3 (0.6)	4	3.87 (0.78)	1.3	2.3	23.2	54.4	18.8	26.8 (23.0–30.9)
Make sure you find the right place to get the health …	476	5 (1.1)	4	3.80 (0.75)	0.6	4.0	24.8	56.3	14.3	29.4 (25.5–33.7)
Find out what healthcare services you are entitled to …	475	6 (1.3)	4	3.47 (0.89)	1.5	11.2	38.3	37.3	11.8	50.9 (46.4–55.4)
Work out what is the best care for you	473	8 (1.7)	4	3.69 (0.77)	1.1	3.8	31.7	51.8	11.6	36.6 (32.3–41.0)
				3.69 (0.61)						36.0
8. Ability to find good health information										
Find information about health problems	479	2 (0.4)	4	4.04 (0.70)	1.0	1.5	11.9	63.9	21.7	14.4 (11.5–17.9)
Find health information from several different places	477	4 (0.8)	4	3.98 (0.81)	1.9	2.7	14.0	58.3	23.1	18.7 (15.4–22.4)
Get information about health so you are up to date …	477	4 (0.8)	4	3.98 (0.71)	1.7	1.3	12.8	66.2	18.0	15.7 (12.7–19.3)
Get health information in words you understand …	475	6 (1.3)	4	3.92 (0.75)	0.8	3.8	16.0	60.8	18.5	20.6 (17.2–24.5)
Get health information by yourself	477	4 (0.8)	4	4.01 (0.77)	1.9	2.3	10.9	62.5	22.4	15.1 (12.1–18.6)
				3.99 (0.61)						16.9
9. Understanding health information well enough to know what to do										
Confidently fill medical forms in the correct way	478	3 (0.6)	4	3.93 (0.83)	1.9	3.3	16.9	55.9	22,0	22.2 (18.7–26.1)
Accurately follow the instructions from healthcare providers	477	4 (0.8)	4	3.93 (0.64)	0.4	1.0	18.7	65.2	14.7	20.1 (16.8–24.0)
Read and understand written health information	476	5 (1.1)	4	4.02 (0.74)	0.8	2.3	14.1	59.2	23.5	17.2 (14.1–20.9)
Read and understand all the information on medication labels	477	4 (0.8)	4	3.81 (0.80)	1.5	3.8	22.9	55.8	16.1	28.1 (24.2–32.3)
Understand what healthcare providers are asking you to do	477	4 (0.8)	4	4.08 (0.71)	1.0	1.7	10.1	63.1	24.1	12.8 (10.1–16.1)
				3.95 (0.56)						20.1

* For scales with agree/disagree response options (scales 1–5), the ‘difficulty’ level was calculated as the proportion responding disagree and strongly disagree as against agree or strongly agree. For the competency scales (scales 6–9), difficulty was calculated as the proportion responding cannot do, very difficult or quite difficult as against quite easy and very easy

**Table 3:** Psychometric properties of the HLQ, Danish version

Item	Factor loading (95 % CI)		R^2^	Composite reliability (95 % CI) Cronbach’s α (italics)
Feeling understood and supported by healthcare providers				0.84 (0.82–0.87); *0.83*
I have at least one healthcare provider who knows me well	0.80	0.77–0.84	0.65	
I have at least one healthcare provider I can discuss …	0.91	0.88–0.94	0.83	
I have the healthcare providers I need to help me work …	0.75	0.71–0.79	0.56	
I can rely on at least one healthcare provider.	0.83	0.80–0.86	0.69	
Fit with 1 correlated residual = χ_WLSMV_^2^ (1) = 6.504, *p* = 0.0108, CFI = 0.999, TLI = 0.980, RMSEA = 0.107 (0.041–0.191)				
Having sufficient information to manage my health				0.84 (0.82–0.87); *0.84*
I feel I have good information about health	0.76	0.71–0.80	0.57	
I have enough information to help me deal with my …	0.83	0.79–0.87	0.69	
I am sure I have all the information I need to manage …	0.89	0.85–0.92	0.79	
I have all the information I need to look after my health	0.85	0.81–0.88	0.72	
Model fit—χ_WLSMV_^2^ (2) = 4.753, *p* = 0.093, CFI = 0.999, TLI = 0.997, RMSEA = 0.054 (0.000–0.118)				
Actively managing my health				0.83 (0.80–0.85); *0.82*
I spend quite a lot of time actively managing my health	0.72	0.67–0.77	0.51	
I make plans for what I need to do to be healthy.	0.93	0.90–0.96	0.87	
Despite other things in my life, I make time to be healthy	0.66	0.60–0.71	0.43	
I set my own goals about health and fitness	0.63	0.58–0.69	0.40	
There are things that I do regularly to make myself more healthy	0.79	0.74–0.84	0.62	
Model fit—χ_WLSMV_^2^ (5) = 18.527, *p* = 0.0024, CFI = 0.994, TLI = 0.988, RMSEA = 0.075 (0.041–0.113)				
Social support for health				0.82 (0.79–0.84); *0.81*
I can get access to several people who understand and …	0.78	0.74–0.83	0.61	
When I feel ill, the people around me really understand …	0.60	0.54–0.67	0.36	
If I need help, I have plenty of people I can rely on.	0.86	0.82–0.89	0.73	
I have at least one person who can come to medical …	0.73	0.68–0.78	0.53	
I have strong support from family or friends.	0.82	0.77–0.86	0.67	
Fit with two correlated residuals = χ_WLSMV_^2^ (3) = 3.101, *p* = 0.3764, CFI = 1.000, TLI = 1.000, RMSEA = 0.008 (0.000–0.078)				
Appraisal of health information				0.77 (0.73–0.80); *0.76*
I compare health information from different sources	0.71	0.65–0.76	0.50	
When I see new information about health, I check up …	0.75	0.70–0.80	0.57	
I always compare health information from different sources …	0.86	0.81–0.90	0.73	
I know how to find out if the health information I receive is …	0.49	0.41–0.56	0.24	
I ask healthcare providers about the quality of the …	0.59	0.52–0.66	0.35	
Fit with one correlated residual = χ_WLSMV_^2^ (4) = 7.509, *p* = 0.1113, CFI = 0.997, TLI = 0.994, RMSEA = 0.043 (0.000–0.090)				
Ability to actively engage with healthcare providers				0.84 (0.81–0.86); *0.84*
Make sure that healthcare providers understand your …	0.60	0.54–0.66	0.36	
Feel able to discuss your health concerns with a …	0.78	0.74–0.83	0.62	
Have good discussions about your health with doctors	0.75	0.70–0.80	0.56	
Discuss things with healthcare providers until you understand …	0.86	0.82–0.89	0.73	
Ask healthcare providers questions to get the …	0.87	0.84–0.91	0.76	
Fit with one correlated residual = χ_WLSMV_^2^ (4) = 5.976, *p* = 0.2010, CFI = 0.999, TLI = 0.998, RMSEA = 0.032 (0.000–0.082)				
Navigating the healthcare system				0.84 (0.82–0.87); *0.84*
Find the right healthcare	0.72	0.66–0.77	0.51	
Get to see the healthcare providers I need to	0.50	0.42–0.57	0.25	
Decide which healthcare provider you need to see.	0.84	0.80–0.88	0.70	
Make sure you find the right place to get the health …	0.85	0.82–0.89	0.73	
Find out what healthcare services you are entitled to	0.78	0.73–0.82	0.61	
Work out what is the best care for you	0.79	0.75–0.83	0.62	
Fit with two correlated residuals = χ_WLSMV_^2^ (7) = 11.293, *p* = 0.1264, CFI = 0.999, TLI = 0.997, RMSEA = 0.036 (0.000–0.072)				
Ability to find good health information				0.87 (0.85–0.89); *0.87*
Find information about health problems	0.81	0.77–0.85	0.65	
Find health information from several different places	0.86	0.83–0.90	0.74	
Get information about health so you are up to date …	0.83	0.79–0.86	0.68	
Get health information in words you understand	0.71	0.67–0.76	0.51	
Get health information by yourself	0.88	0.85–0.91	0.77	
Model fit—χ_WLSMV_^2^ (5) = 5.106, *p* = 0.4031, CFI = 1.0, TLI = 1.0, RMSEA = 0.007 (0.000–0.064)				
Understanding health information well enough to know what to do				0.81 (0.79–0.84); *0.81*
Confidently fill medical forms in the correct way	0.69	0.64–0.75	0.48	
Accurately follow the instructions from healthcare providers	0.69	0.63–0.75	0.48	
Read and understand written health information	0.83	0.79–0.87	0.68	
Read and understand all the information on medication labels	0.80	0.76–0.84	0.65	
Understand what healthcare providers are asking you to do	0.78	0.73–0.83	0.61	
Fit with one correlated residual = χ_WLSMV_^2^ (4) = 5.897, *p* = 0.2070, CFI = 0.999, TLI = 0.998, RMSEA = 0.031 (0.000–0.081)				

The model fit for all scales (with some inclusion of correlated errors related mostly to linguistic overlap) was generally very good, demonstrating that the scales are homogeneous, although, after model modification, the RMSEA remained unacceptably high for scale 1 (Table 3) suggesting some further association between the item residuals in this scale. Initially four scales (scales 2, 3, 7 and 8) returned a satisfactory close fit for the one-factor models. For five scales, the close fit statistics were initially not satisfactory due to the presence of correlated residuals which, when included in the model (maximum 2), ranged from 0.23 (scale 4) to 0.49 (scale 1).

For each scale there were high loadings on almost all items [all above 0.6 except for one each in scale 5 (0.49) and scale 7 (0.50)]. The median loading was 0.79. A composite reliability and Cronbach’s α of ≥0.8 was observed for all scales except scale 5. ‘Appraisal of health information’ (composite reliability = 0.77, α = 0.76). The median composite reliability was 0.84 (α = 0.83), with the highest for scale 8. ‘Ability to find good health information’ (composite reliability = 0.87, α = 0.87). In summary, the reliability for all scales was good (0.8–0.9) except for scale 4. These findings are in the same range as what was observed in the original psychometric studies of the English HLQ.

A nine-factor CFA model was fitted to the 44 items with no cross-loadings or correlated residuals allowed. Given the very restricted nature of the model, the fit was quite satisfactory: *χ*_WLSMV_^2^ (866 df) = 2459.31, *p* < 0.0001, CFI = 0.934, TLI = 0.930 and RMSEA = 0.062. While the CFI and TLI are lower than the pre-specified cut-off, this is not surprising given the large number of parameters in the model set precisely to 0.0. Also, the CFI and TLI tend to underestimate the goodness-of-fit of models with large numbers of measured variables compared with the RMSEA as in this analysis (Kenny and McCoach 2003). The ranges of the factor loadings in this model were: scale 1. ‘Feeling understood and supported by healthcare providers’: 0.78–0.95; scale 2. ‘Having sufficient information to manage my health’: 0.77–0.86; scale 3. ‘Actively managing my health’: 0.69–0.89; scale 4. ‘Social support for health’: 0.62–0.84; scale 5. ‘Appraisal of health information’: 0.58–0.83; scale 6. ‘Ability to actively engage with healthcare providers’: 0.69–0.90; scale 7. ‘Navigating the healthcare system’: 0.57–0.81; scale 8. ‘Ability to find good health information’: 0.79–0.89; and scale 9. ‘Understanding health information well enough to know what to do’: 0.69–0.86.

Inter-factor correlations in the nine-factor model ranged from 0.41 (scales 4 and 9) to 0.93 (scales 8 and 9). Inter-factor correlations were >0.80 for scales 6/7 = 0.82, 7/8 = 0.87, 6/9 = 0.84, 7/9 = 0.92 and 8/9 = 0.93. It is frequently argued that an inter-factor correlation of >0.80 to >0.85 indicates a potential lack of discriminant validity (Brown 2006). This suggests that there may be a lack of discriminant validity in second part of the HLQ, particularly for scales 7, 8 and 9.

By allowing some flexibility in the estimation of residual correlations and cross-loadings the nine-factor BSEM model was an excellent fit to the data (the posterior predictive probability value was 0.49 compared with the target value of 0.50). There were nine statistically significant cross-loadings, the largest being 0.28. Correlations between scales 6–9 were 6/7 = 0.72, 6/8 = 0.63, 6/9 = 0.67, 7/8 = 0.83, 7/9 = 0.85, 8/9 = 0.84.

Table 4 shows pattern of HLQ scores according to sociodemographic variables. Females were somewhat higher than males only on 8. ‘Ability to find good health information’. Younger people had substantially higher scores on 4. ‘Social support for health’ than older people. There were differences on six HLQ variables for education, all indicating that people with higher education have higher health literacy than those with less education. The strongest effects were seen for 8. ‘Ability to find good health information’. There are no significant differences between those who did or didn’t have Danish as their mother tongue. Five scales showed that those with a long term illness or disability had higher health literacy, the largest effect was seen for 4. ‘Social support for health’ and 2. ‘Having sufficient information to manage my health’. The strongest pattern of all exogenous variables was with self-rated health. All scales demonstrated a difference between those rating themselves as having 'Excellent' or 'Very good' health compared with 'Good' or worse health . The effect was strongest for 4. ‘Social support for health’, 2. ‘Having sufficient information to manage my health’, and 3. ‘Actively managing my health’.

**Table 4:** Relationships between sociodemographic variables and HLQ scores

Variable	1. Feeling understood and supported by healthcare providers	2. Having sufficient information to manage my health	3. Actively managing my health	4. Social support for health	5. Appraisal of health information	6. Ability to actively engage with healthcare providers	7. Navigating the healthcare system	8. Ability to find good health information	9. Understand health information enough to know what to do
*Sex*									
Female									
Mean (SD)	3.00 (0.59)	3.00 (2.53)	2.85 (0.50)	3.15 (0.56)	2.77 (0.54)	3.98 (0.60)	3.71 (0.60)	*4.05* (0.59)	3.99 (0.51)
Male									
Mean (SD)	3.00 (0.61)	2.99 (0.54)	2.82 (0.62)	3.13 (0.52)	2.72 (0.58)	4.01 (0.58)	3.67 (0.62)	*3.91* (0.63)	2.91 (0.62)
ANOVA	F = 0.00; 1, 473 df; *p* = 0.950	F = 0.12; 1, 472 df; *p* = 0.732	F = 0.28; 1, 469 df; *p* = 0.600	F = 0.19; 1, 474 df; *p* = 0.666	F = 0.86; 1, 473 df; *p* = 0.354	F = 0.33; 1, 473 df; *p* = 0.569	F = 0.47; 1, 466 df; *p* = 0.493	F = 6.32; 1, 469 df; *p* = 0.012	F = 2.91; 1, 470 df; *p* = 0.090
ES (95 % CI)	0.01 (−0.18 to 0.19)	0.04 (−0.23 to 0.14)	0.05 (−0.13 to 0.23)	0.04 (−0.14 to 0.22)	0.09 (−0.10 to 0.27)	−0.05 (−0.24 to 0.13)	0.06 (−0.12 to 0.25)	*0.24* (0.05 to 0.42)	0.16 (−0.02 to 0.34)
*Age*									
<65 years									
Mean (SD)	3.00 (0. 62)	3.05 (0.54)	2.84 (0.56)	*3.20* (0.50)	2.77 (0.54)	3.97 (0.59)	3.69 (0.60)	4.01 (0.57)	3.95 (0.55)
≥65 years									
Mean (SD)	3.01 (0.53)	3.08 (0.52)	2.83 (0.51)	*2.99* (0.55)	2.66 (0.60)	4.02 (0.55)	3.73 (0.61)	3.94 (0.67)	3.99 (0.61)
ANOVA	F = 0.01; 1, 444 df; *p* = 0.923	F = 0.30; 1, 442 df; *p* = 0.586	F = 0.02; 1, 439 df; *p* = 0.879	F = 12.50; 1, 444 df; *p* > 0.001	F = 3.55; 1, 443 df; *p* = 0.060	F = 0.65; 1, 442 df; *p* = 0.421	F = 0.35; 1, 436 df; *p* = 0.556	F = 1.28; 1, 439 df; *p* = 0.258	F = 0.41; 1, 440 df; *p* = 0.521
ES (95 % CI)	−0.01 (−0.23 to 0.21)	−0.06 (−0.28 to 0.16)	−0.02 (−0.21 to 0.24)	*0.40* (0.18 to 0.62)	0.21 (−0.01 to 0.44)	−0.09 (−0.31 to 0.13)	−0.07 (−0.29 to 0.16)	0.13 (−0.09 to 0.35)	−0.07 (−0.30 to 0.15)
*Education*									
≤Year 12									
Mean (SD)	2.98 (0.56)	*2.97* (0.55)	2.77 (0.57)	*3.08* (0.53)	*2.64* (0.56)	3.95 (0.61)	*3.59* (0.65)	*3.83* (0.67)	*3.86* (0.61)
High school diploma									
Mean (SD)	3.03 (0.62)	*3.13* (0.53)	2.88 (0.55)	*3.20* (0.51)	*2.82* (0.56)	4.02 (0.57)	*3.78* (0.56)	*4.13* (0.51)	*4.04* (0.51)
ANOVA	F = 0.78; 1, 442 df; *p* = 0.379	F = 9.61; 1, 440 df; *p* = 0.002	F = 3.72; 1, 437 df; *p* = 0.054	F = 5.72; 1, 442 df; *p* = 0.017	F = 10.38; 1, 440 df; *p* = 0.001	F = 1.57; 1, 440 df; *p* = 0.210	F = 10.49; 1, 434 df; *p* = 0.001	F = 28.19; 1, 437 df; *p* = 0.000	F = 11.75; 1, 438 df; *p* = 0.001
ES (95 % CI)	−0.08 (−0.27 to 0.10)	−*0.30* (−0.48 to −0.11)	−0.19 (−0.37 to −0.00)	−*0.23* (−0.42 to −0.04)	−*0.31* (−0.50 to −0.12)	−0.12 (−0.31 to 0.07)	−*0.31* (−0.50 to −0.12)	−*0.51* (−0.70 to −0.32)	−*0.33* (−0.52 to −0.14)
*Mother tongue*									
Danish									
Mean (SD)	3.00 (0.59)	3.06 (0.54)	2.83 (0.56)	3.14 (0.53)	2.73 (0.56)	3.98 (0.59)	3.69 (0.60)	3.99 (0.59)	3.95 (0.56)
Other									
Mean (SD)	2.99 (0.67)	3.04 (0.53)	2.85 (0.51)	3.09 (0.42)	2.89 (0.53)	3.99 (0.62)	3.71 (0.69)	4.13 (0.67)	4.06 (0.59)
ANOVA	F = 0.01; 1, 472 df; *p* = 0.936	F = 0.03; 1, 471 df; *p* = 0.548	F = 0.04; 1, 640.6 df; *p* = 0.835	F = 0.34; 1, 473 df; *p* = 0.558	F = 2.51; 1, 472 df; *p* = 0.114	F = 0.01; 1, 471 df; *p* = 0.931	F = 0.02; 1, 465 df; *p* = 0.893	F = 1.81; 1, 468 df; *p* = 0.180	F = 1.30; 1, 469 df; *p* = 0.255
ES (95 % CI)	0.01 (−0.34 to 0.37)	0.03 (−0.32 to 0.38)	−0.04 (−0.39 to 0.32)	0.10 (−0.24 to 0.45)	−0.28 (−0.63 to −0.07)	−0.02 (−0.36 to 0.33)	−0.02 (−037 to 0.33)	−0.24 (−0.59 to 0.11)	−0.20 (−0.55 to 0.15)
*Long illness or disability*									
Yes									
Mean (SD)	2.97 (0.59)	*3.13* (0.49)	*2.90* (0.54)	*3.22* (0.45)	*2.80* (0.53)	3.99 (0.59)	3.73 (0.60)	*4.04* (0.58)	3.98 (0.54)
No									
Mean (SD)	2.92 (0.58)	*2.94* (0.60)	*2.74* (0.55)	*3.02* (0.58)	*2.67* (0.59)	3.98 (0.62)	3.63 (0.61)	*3.92* (0.63)	3.93 (0.58)
ANOVA	F = 2.23; 1, 474 df; *p* = 0.136	F = 13.95; 1, 473 df; *p* = 0.000	F = 8.85; 1, 640.6 df; *p* = 0.003	F = 17.78; 1, 475 df; *p* = 0.000	F = 6.47; 1, 473 df; *p* = 0.011	F = 0.02; 1, 473 df; *p* = 0.900	F = 2.58; 1, 467 df; *p* = 0.109	F = 4.32; 1, 470 df; *p* = 0.038	F = 0.96; 1, 471 df; *p* = 0.328
ES (95 % CI)	−0.14 (−0.32 to 0.04)	*0.35* (0.16 to 0.53)	*0.28* (0.09 to 0.46)	*0.39* (0.21 to 0.58)	*0.24* (0.05 to 0.42)	0.01 (−0.17 to 0.19)	0.15 (−0.03 to 0.34)	*0.20* (0.01 to 0.38)	0.09 (−0.09 to 0.28)
*Self-rated health*									
Excellent, very good									
Mean (SD)	*3.06* (0.60)	*3.17* (0.50)	*2.94* (0.53)	*3.26* (0.47)	*2.84* (0.53)	*4.05* (0.54)	*3.79* (0.60)	*4.10* (0.57)	*4.04* (0.53)
Good, less good, poor									
Mean (SD)	*2.92* (0.58)	*2.91* (0.60)	*270* (0.55)	*2.99* (0.54)	*2.64* (0.57)	*3.92* (0.60)	*3.56* (0.61)	*3.85* (0.62)	*3.95* (0.58)
ANOVA	F = 6.33; 1, 474 df; *p* = 0.012	F = 27.67; 1, 473 df; *p* = 0.000	F = 23.98; 1, 470 df; *p* = 0.000	F = 32.01; 1, 475 df; *p* = 0.000	F = 15.58; 1, 473 df; *p* = 0.000	F = 5.81; 1, 473 df; *p* = 0.016	F = 18.30; 1, 467 df; *p* = 0.000	F = 19.80; 1, 470 df; *p* = 0.000	F = 14.17; 1, 471 df; *p* = 0.000
ES (95 % CI)	*0.23* (0.05 to 0.41)	*0.49* (0.30 to 0.67)	*0.45* (0.27 to 0.64)	*0.52* (0.34 to 0.70)	*0.36* (0.18 to 0.55)	*0.22* (0.04 to 0.40)	*0.40* (0.21 to 0.58)	*0.41* (0.23 to 0.60)	*0.35* (0.17 to 0.53)

*ES* effect size

## Discussion

In this study, we applied rigorous linguistic and cultural adaption methods to the HLQ to produce a high quality Danish version. We used a process we call measurement adaptation whereby the meanings of the original concepts were carefully reproduced. Consequently, data from a diverse validation sample of the Danish population demonstrated that the translated HLQ has a strong psychometric structure, and the reliability of the original HLQ was maintained. Within each scale, the Danish items have a range of difficulty that should enable the Danish HLQ to be sensitive to differences between groups and change over time. Importantly, respondents clearly understood all the translated items. This series of rigorous psychometric and practical tests indicate that the Danish HLQ is robust and suitable for application in Danish settings, and that the reproduction of the nine scales in a different language and setting attests to the fundamental separate elements embodied in each of the HLQ constructs (Osborne et al. 2013).

The cognitive interviews generated valuable information. It was demonstrated that the translated items were understood according to the item intents by Danish people from a wide range of backgrounds.

Within scales there was some item residual correlation. Such correlations can mean construct complexity (i.e., sub-constructs) or item redundancy (Boyle 1991). In some cases, this seemed to be due to limited choices of words in the Danish language. For example, two separate words exist in English for ‘asking’ and ‘discussing’ (items 6.4 and 6.5) but not in Danish in this context. For items 5.1 and 5.3, the correlation is thought to relate to the positioning of these items on Bloom’s taxonomy (Amer 2006). That is, the items measure the easy and difficult ends of the same dimension: item 5.1 (0.27) is a relatively easy item on which to score highly, and item 5.3 (0.41) is a relatively hard item. While there is some conceptual overlap across these items, this scale, like most HLQ scales, has a good range of item strength and therefore has wide coverage of the target construct.

The majority of the Danish HLQ items load highly on their respective factors and the scales have good reliability. The fit of both single-factor and nine-factor models to the data was generally good, indicating scale homogeneity. Further, scales 1–6 showed clear discriminant validity, while discriminant validity was not clearly established for scales 7–9. By allowing for small correlated item residuals and cross-loadings the BSEM analysis showed inter-factor correlations among scales 6–9 that were somewhat smaller, however three remained >0.80 but ≤0.85. The overlap between scales 7–9 suggests that there may be a higher-order factor or causal linkages determining the stronger association between the concepts measured by these scales. Strong associations between these scales have been noted in previous analyses of the English HLQ (Osborne et al. 2013). Scales 8 and 9 focus on the ability to locate (scale 8) and appraise (scale 9) health information, while scale 7 (health system navigation) may be seen as a closely linked outcome of these abilities.

Every item clearly loaded on its own factor, with only three of the 44 items loading <0.6. Given that every scale comprised only four, five or six items, this demonstrates parsimony: that is, a minimum number of items are administered to respondents to reliably capture the full breadth of the intended constructs (Boyle 1991). Many questionnaires achieve high reliability through the inclusion of large numbers of items, or of items that have only minor linguistic differences and conceptual redundancy (Boyle 1991). In the Danish HLQ, only one scale had reliability below our nominal 0.8 cut-off: 5. ‘Appraisal of health information’ (0.77). This scale included the most difficult items and asked respondents about a range of challenging tasks such as comparing, checking up and finding out about information. These concepts are high in Bloom’s taxonomy (Krathwohl 2002; Amer 2006) and are likely to be more difficult for respondents to attend to and process successfully. Thus respondents may have found it challenging to judge their own critical appraisal ability, and this possibly contributed to the lower, but acceptable, reliability of the scale.

The wide ranging difficulty of items within a scale was an important and deliberate step in the development of the HLQ as it is intended to make the questionnaire sensitive to small differences at both the low score (e.g., strongly disagree or cannot do) and high score (e.g., strongly agree or very easy) ends of the scales. The ability to measure differences between subgroups was demonstrated in a recent study that used two of the scales from the Danish HLQ (scales 6 and 9—the understanding and engagement dimensions of health literacy) (Bo et al. 2014). The response options were slightly different because the extreme cannot do category in this general survey was omitted. However, the estimates clearly pointed out that 8–20 % of the Danish population have difficulties with these two dimensions of health literacy. Further, the study showed that the elderly, immigrants, those with limited formal education, and low-income groups were more likely to have limited health literacy skills. The findings are in line with Dutch results using the European Health Literacy Survey, where having a low level of education, or a low perceived social status, or being male were found to be modestly related to low health literacy scores, mainly for accessing and understanding health information (van der Heide et al. 2013).

The HLQ scales were related to the sociodemographic subgroups in our sample in reasonably expected ways. While the items were written specifically to avoid potential bias in gender, age and education, the data indicated that women were slightly better than men in their ability to find good information, however differences were not observed in other domains. Younger people (<65 years), compared with older people (≥65 years), reported more ‘Social support for health’ (scale 4), probably related to isolation in older people. As expected, education was clearly related to health literacy, but primarily in those HLQ domains with content covering finding and processing information (scales 2, 5, 7, 8 and 9) and not with domains related to feeling understood or engaging with health care professionals (scales 1, 3 and 6). In previous research we have seen language variables, such as the language spoken at home, to be strongly related to health literacy (Beauchamp et al. 2015). In the current study, with 93 % having Danish as their mother tongue and where no questionnaires were offered in other languages, almost no association between language and health literacy was observed. Only a small effect was observed for the scale with content requiring the highest language skills, 5. ‘Appraisal of health information’, where those without Danish reported slightly higher scores. It is possible that this small group included nationalised professionals, however future studies need to explore this in more detail.

Having a long term illness or disability was moderately and positively related to several health literacy domains. This is consistent with the notion that health literacy develops in individuals as they gain experience with managing illnesses, working with practitioners over many years, and through overcoming information barriers (Paasche-Orlow and Wolf 2007). Finally, we expect that good health literacy is a determinant, antecedent or consequence of good health (Paasche-Orlow and Wolf 2007; Berkman et al. 2011; Batterham et al. 2016). Consequently, we observe this pattern through all nine HLQ domains positively related to self-rated health.

### Strengths and limitations

It is intended that the HLQ can be used in diverse settings, and with people with broad-reaching sociodemographic profiles and various health conditions. A strength of this study is that the validation sample was drawn from communities across a wide range of locations, with an array of health conditions, and who were young and old. The face-to-face administration of the questionnaire ensured a high participation rate, particularly from people with limited reading and writing abilities who usually are excluded from taking part in questionnaire studies.

There are several indications that the Danish HLQ has good psychometric properties. The use of modern psychometric procedures permitted the application of highly demanding tests, and the analysis has clearly shown that the HLQ multi-dimensional construct of health literacy comprises nine separate and cogent scales. A nine-factor CFA model demonstrated good construct validity. A model with no residual correlations or cross-loadings was an acceptable fit to the data while a very good fit was achieved with a BSEM analysis that allowed modest correlated residuals and cross-loadings.

The stringent linguistic, cultural and measurement adaptation procedures are likely to have contributed to the strong psychometric performance of the Danish HLQ. This procedure includes an extensive item intent document that explains what the elements of individual items mean and do not mean. This document, along with the intensive translation consensus meeting, ensures the translators are supported to capture the precise meaning, context, difficulty, and measurement juxtaposition of one item with other items within each scale.

A strength of the HLQ is that it has sensitivity to group differences related to illness or disability, and self-rated health. This has also been demonstrated in other settings (Beauchamp et al. 2015; Zhang et al. 2016; Vamos et al. 2016). While it is important that differences between groups are demonstrated, research needs to be done to demonstrate sensitivity to change. This issue is being addressed in a range of ongoing intervention studies where the HLQ is part of the assessments of a range of outcomes (Batterham et al. 2014; Livingston et al. 2014; Redfern et al. 2014; Banbury et al. 2014; Barker et al. 2015; Griva et al. 2015).

Another possible limitation that needs further investigation is the somewhat high inter-correlations between Danish HLQ scales 7, 8 and 9. These data suggest that there may be a lack of discriminant validity between these scales; however, this may also be explained by higher-order models and causal relationships between these constructs. This provides guidance for further work, particularly in exploring how the scales respond, with careful exploration of rates of change (or no change) over time.

Finally, this study only explored administration of the HLQ in face-to-face interviews. This is a strength of the study because it overcomes illiteracy of respondents, but future work should continue to explore other modes of administration including self-administered written formats, and confirm whether the HLQ produces valid information that results in improved services across the Danish society.

## Conclusion

In conclusion, this study demonstrates that the Danish HLQ has strong construct and content validity, and high composite reliability. As such, the HLQ is now available for application in Denmark with nine scales providing a robust multi-dimensional approach to understanding health literacy. It is incumbent on the developers of questionnaires to demonstrate the measurement properties of new questionnaires, and that the data they return to consumers, practitioners, policymakers and researchers provide valid inferences and are reliable. The findings of this study may be an important contribution to health literacy research, and the data support the web of evidence of measurement validity of the interpretation of HLQ scores for use in national and international population health and healthcare.

## Abbreviations

BSEM: Bayesian structural equation modelling
CFA: confirmatory factor analysis
CFI: comparative fit index
CI: confidence interval
HLQ: Health Literacy Questionnaire
HLS-EU: European Health Literacy Survey
IQR: inter quartile range
MI: modification indices
RMSEA: root mean square error of approximation
SD: standard deviation
SEPC: standardised expected parameter change
TLI: Tucker–Lewis index
WLSMV: weighted least squares mean and variance adjusted
